# Proteomic analysis of aqueous humor from patients with myopia

**Published:** 2008-03-03

**Authors:** Xiaoming Duan, Qingjun Lu, Peng Xue, Hongjie Zhang, Zhe Dong, Fuquan Yang, Ningli Wang

**Affiliations:** 1 Beijing Tongren Eye Center, Beijing Tongren Hospital, Capital Medical University; 2 Beijing Institute of Ophthalmology, Beijing Tongren Eye Center, Beijing Tongren Hospital, Capital Medical University; 3 Protein Research Platform, Chinese Academy of Science, Beijing, China

## Abstract

**Purpose:**

The mechanism of axial elongation in the myopic eyeball remains to be elucidated. It is known that the expression profile for some proteins in the aqueous humor (AH) changes in some diseases. Accordingly, determinations of these AH proteins may serve to understand their potential role in this pathogenesis. To identify the possible mechanism in myopia development, a proteomic analysis of the AH composition from high myopic eyes (patients) was performed and compared with that of the AH composition from non-myopic cataract eyes (controls).

**Methods:**

Total protein concentration in AH was determined by the Bradford method, and separation profiles were analyzed by two-dimensional (2D) gel electrophoresis. Protein in gel was determined by silver stain, and the separation profiles were analyzed to assess spot density changes between myopia and non-myopia patients. These spots in gel were isolated and identified by mass spectrometry.

**Results:**

The total protein concentration in AH with high myopia was significantly greater than that of non-myopia. A total of six spots were significantly increased in 2D gels from high myopia. The spots were derived from albumin, transthyretin, and a vitamin D-binding protein.

**Conclusions:**

The protein composition in AH was significantly different between myopia and non-myopia. The identified proteins could be a potential biomarker for high myopia development and may play a role in the mechanisms of myopia ocular axial elongation.

## Introduction

Myopia is the most common eye disease in the world with substantial social, educational, and economic impact [1]. The prevalence of high myopia (usually defined as eyes with ≥6 diopters [D] of myopia or ≥26 mm in length) in world populations has been estimated to be between 0.3% and 9.6%. However, this percentage has recently been shown to be as high as 16% in certain young Asian populations, and evidence suggests that this prevalence is increasing [2]. It is well documented that individuals with high myopia have a greatly increased risk of ocular pathology such as retinal detachment, macular degeneration, and glaucoma [3,4]. However, the pathogenesis of high myopia is not well understood. Previous studies have focused upon investigating changes in the sclera, retina, and choroid in high myopia, but few studies have been directed toward assessing the aqueous humor (AH) in these patients. It was reported that the pigment epithelium–derived factor (PEDF) is elevated in patients with high myopia [5]. Such findings suggest that some proteins in AH might be involved in the development of high myopia.

AH is an important intraocular fluid responsible for the supply of nutrients to and removal of metabolic wastes from the avascular tissues of the eye [6]. It contains proteins secreted from anterior segment tissues, and these proteins could play a role in the pathogenesis of various eye diseases [7]. It is known that protein levels in AH are changed in various eye diseases. Not only are such changes in AH proteins observed in anterior segment disorders but also in posterior segment disorders [5,8-11]. In addition, an increasing number of studies have demonstrated that some proteins that changed in AH correlate with the mechanisms or prognosis of many eye disorders [12-14].

In this report, we used proteomics as a means to identify disease-specific proteins in AH. Through comparative analyses of the proteomes in cataract (control) and high myopia (patients), it is possible to achieve a better understanding of the molecular events involved in myopia development and generate essential data needed for elaborating more effective strategies with regard to identification of new biomarkers and/or treatments. The proteomic techniques used include protein separation by two-dimensional gel electrophoresis (2-DE) and characterization by mass spectrometry of peptides, amino acid sequencing, and bioinformatics analysis. High resolution two-dimensional (2D) polyacrylamide gel electrophoresis (PAGE) is a technique for analyzing several hundred proteins in tissues, fluids, or cells using only a few microliters of sample and is therefore theoretically ideal for analyzing limited volumes of AH. Funding et al. [15] have used the method to find the different proteins in AH from patients with acute corneal rejection. Accordingly, this platform has been widely used to unravel and identify critical changes in myopia-related proteins.

In this study, we investigated the differential proteomes in five high myopias (patients) and matched cataracts (controls). Abnormal expressions and distributions of proteins from AH were identified and evaluated in age-paired clinical specimens.

## Methods

### Sample collection

AH samples were collected at Beijing Tongren Eye Center, Capital Medical University, Beijing, China. Informed consent was obtained from all patients. During the period from October 2005 to May 2006, AH samples were collected from five patients with high myopia (patients) and from five age-matched cataract patients without high myopia (controls). Patients with axial length ≥26 mm were included as high myopia [16]. Axial lengths were measured by ophthalmology ultrasonic diagnosis apparatus (Quantel Medical, Echography, AXIS-II; Clermont-Ferrand, Auvergne region, France). The control cases were determined as having normal intraocular pressure (IOP; of less than 20 mmHg in at least two measurements separated by more than one day) and were undergoing routine senile cataract surgery for visual rehabilitation.

Case exemptions included patients with other ophthalmic diseases (for example, glaucoma, uveitis, progressive retinal disease), systemic diseases (for example, diabetes mellitus, arthritis), and patients who received local medications or had undergone previous laser or intraocular surgery.

All AH samples were obtained before ocular incision during phacoemulsification surgery. Approximately 100-200 μl AH was collected by a 26 gauge ophthalmic cannula under a binocular microscope. The AH was immediately stored in aliquots of 50 μl in 1.5 ml microtubes at −80 °C until further analysis.

### Total protein quantization

Total AH protein concentration was determined according to the Bradford method (Bio-Rad Laboratories, Hercules CA) following the manufacturer’s protocol. Bovine serum albumin (BSA) was used to create a standard reference. AH (2 μl) from each sample was added to 18 μl distilled water and mixed with 200 μl of dye from the Bradford reagent. After being incubated for 5 min at room temperature, absorbances at 595 nm optic light were detected by the fluorophotometer (DU800; Beckman Coulter, Inc, Fullerton, CA). The protein concentration from each sample was calculated according to the linearized BSA absorbance curve.

### Two-dimensional gel electrophoresis

The principle procedure for running 2D gels has been described in detail previously [15]. The AH samples (45 μl) were solubilized directly in 55 μl lysis solution (8.0 M urea, 4% 3,3-cholamidopropyl-dimethylammonio-1-propanesulfonate [CHAPS], 0.5% IPG-buffer, pH 3–10) and 100 μl rehydration solution (8.0 M urea, 2% CHAPS, 1% IPG-buffer,  and 0.1 M DTT) to achieve a total volume of 200 μl AH, lysis solution, and rehydration solution. The final solution was used to rehydrate immobilized pH gradient stripes overnight with linear pH 3–10 at a length of 110 mm (Immobilize Drystripes; Amersham Biosciences Inc., Amersham, UK).

For the first dimension, isoelectric focusing was performed on an Ettan Multiphor II flat bed unit with minor modifications of the manufacturer’s description (Amersham Biosciences Inc., Amersham, UK). For the second dimension, samples were separated by PAGE on a separating gel (1 mm thickness, 12% SDS gel; Hoefer SE600 Ruby; Amersham Biosciences Inc., Amersham, UK). The first-dimension gel stripes and the molecular mass marker protein weight with a range of 14.4-97.4 kDa were attached to the gel using 1% agarose. A 20 mA constant current was applied per gel to run the gel until the dye reached the approximate bottom of the gel.

**Table 1:** Data from patients with myopia and from controls without myopia.

**Group**	**Number**	**Age**	**Sex**	**VA**	**Axial length (mm)**	**Cataract**
Patient	1	68	F	CF	30.92	III-IV
	2	49	M	39680	34.75	III
	3	53	F	39498	31.7	III
	4	86	M	39498	26.49	IV
	5	44	M	39527	35.3	II-III

Control	1	73	F	39498	22.79	III-IV
	2	68	M	CF	22.31	III
	3	55	F	39558	22.06	III
	4	83	M	39558	23.02	IV
	5	43	M	39527	23.38	II-III

Ten AH samples were included in this study, five from cataract patients with high myopia and five from age-matched cataract patients without myopia. CF=Count Finger; VA=Visual Acuity.

The separated proteins within the gels were fixed over a 2 h period in a 40% ethanol and 10% acetic acid solution followed by the silver stain of protein in gel according to the manufacturer’s protocol (Silver Stain Plus kit; BioRad).

### Analysis of two-dimensional gels

The stained gels were scanned with an Imagescanner (Amersham Biosciences Inc.) and analyzed with Imagemaster 2D platinum software (Amersham Biosciences Inc.). The gels were matched according to the number of user-defined landmarks on the gels. Each spot was quantified by using the volume and % volume of the stained spot. We chose to work only the spots with ratios that were greater than 2 between % volume in patients and in controls.

### Protein Identification by HPLC ESI-MS/MS

Gel pieces were washed twice in 25 mM ammonium bicarbonate (AmBic) and dried in a SpeedVac for 10 min. Samples were reduced in 10 mM dithiothreitol and 25 mM AmBic for 45 min at 50 °C and then alkylated in 50 mM iodoacetic acid and 25 mM AmBic for 1 h at room temperature in the dark. Gel pieces were then washed twice in 25 mM AmBic and 50% acetonitrile and vacuum-dried. Proteins were proteolysed with 20 ng of modified trypsin (Promega, Madison, WI) in 25 mM AmBic overnight at 37 °C. The supernatant was collected and peptides were further extracted in 0.1% acetic acid and 60% acetonitrile. Peptide extracts were vacuum-dried and resuspended in 20 μl of water for mass analysis. Liquid chromatography/mass spectrometry/mass spectrometry (LC MS/MS) analysis was performed in an LTQ tandem mass spectrometer (ThermoFinnigan, San Jose, CA) coupled online with an HPLC system. Protein digests obtained above were loaded on the HPLC, which was connected with an in-house made fused silica capillary column (10-cm-length; 100 µm inner diameter) and packed with sunchrom 5 μm C18 resin. High voltage (2.55 kV) was applied to the HPLC side of the column through a zero dead volume MicroCross PEEK connector (Upchurch Scientific, Oak Harbor, WA). The peptides were sequentially eluted from the HPLC column with a gradient of 0-90% of Buffer B (acetonitrile:water:acetic acid, 80:9.9:0.1) in Buffer A (acetonitrile:water:acetic acid, 5:94.9:0.1) at a flow rate of approximately 500 nl/min using Voyager pumps. The eluted peptides were sprayed directly from the tip of the capillary column to the LTQ mass spectrometer for mass spectrometry analysis. The LTQ was operated in a data-dependent mode where the machine measured intensity of all peptide ions in the mass range of 400 to 2000 (mass-to-charge ratios) and isolated the peptide peak with the highest intensity for collision-induced dissociation. All tandem spectra were searched against the *H. sapiens* NCBI reference database (human.nci) using SEQUEST (version 2.7). Results were filtered by Xcorr +1>1.9,+2>2.5,+3>3.75, sp>500, Deltcn>0.1, Rsp<=5. Every protein identified matched at least two peptides.

### Statistics

SPSS Version 11.0 software was used to calculate p-values as achieved with the Mann–Whitney test for all data obtained. A p<0.05 was required for the results to be considered statistically significant.

## Results

Ten AH samples were included in this study, five from cataract patients with high myopia (mean age 60±17.07 years; three males and two females) and five from age-matched cataract patients without myopia (mean age 64.4±15.65 years; three males and two females). There was no statistically significant difference between the two groups with regard to age (p=0.675). Clinical data from the 10 patients are summarized in Table 1.

### Protein content in aqueous humor from patients and controls

The mean total protein level in AH from patients with high myopia was 0.6956 mg/ml (range=0.3316-1.0516 mg/ml) while that from controls was 0.3329 mg/ml (range=0.2925-0.3397 mg/ml; Table 2). Total protein levels in patients were significantly greater than that of controls (p=0.009).

**Table 2:** Measurement of total protein in the aqueous humor of patients and controls.

**Sample**	**Total protein (mg/ml)**
Patients	
1	0.597
2	0.869
3	1.0516
4	0.5577
5	0.4027
Mean	0.6956

Controls	
1	0.3396
2	0.2936
3	0.3991
4	0.2925
5	0.3397
Mean	0.3329
p-value	0.009

The mean total protein level in AH from patients with high myopia was 0.6956 mg/ml (Range = 0.3316~1.0516 mg/ml) while that from controls was 0.3329 mg/ml (Range = 0.2925~0.3397 mg/ml).

### Two-dimensional gel electrophoresis patterns

**Figure 1:**
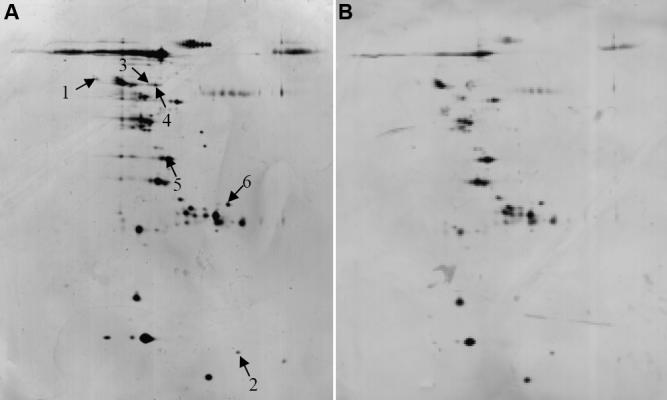
Silver-stained two-dimensional gels of the aqueous humor. **A **and **B** shows a representative gel from a patient (patient number 4 in Table1) and from a control subject (control number 4 in Table1). Total protein concentration in AH was 0.5577 mg/ml from the patient and 0.2925 mg/ml from the control. Arrows and numbers showed six spots with volumes significantly increased by values greater than twofold in the patients. The identities of the spots were derived from vitamin D binding protein (1), tranthyretin (2), and albumin (3, 4, 5, and 6).

Figure 1A,B show a representative gel from a patient (patient number 4 in Table 2) and a gel from a control subject (control number 4 in Table 2). Gels from patients displayed more spots and more intensely silver stained spots than in gels from controls (Figure 1B). There were significant differences in relative spot volumes (% volume) in the gel patterns where patients showed greater volumes than controls (Table 3). No protein spots were found to be downregulated in patients compared to the controls.

### Identification of proteins

**Figure 2:**
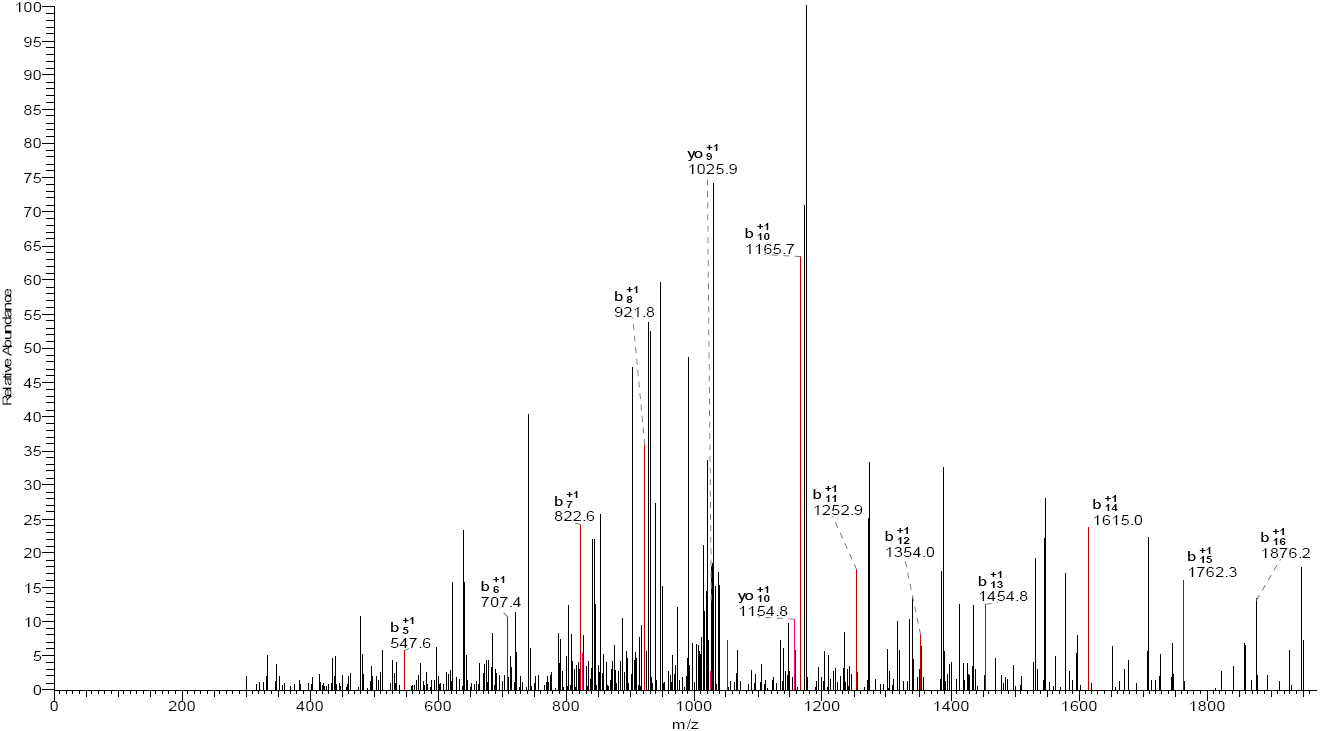
MS/MS spectrum of vitamin D-binding protein derived peptide. The vitamin D-binding protein peptide, SLGECCDVEDSTTCFNAK ([M+H]^2+^=1047.63), was identified using this spectrum, which showed many characteristic y and b series ions.

Based on the above results, six protein spots were isolated for further analysis. Each spot was acquired from the gel and digested extensively with trypsin. The resulting peptides were applied to a LC MS/MS for measurements. For spot 1 (shown to have a significant increase in patients), 10 peptides were captured and measured. Figure 2 shows a typical MS/MS spectrum of its parent ion m/z=1047.63(sequence: SLGECCDVEDSTTCFNAK). Many “y” and “b” series ions from this peptide were clearly identified in this spectrum. Sequence searching indicated that this peptide was from human vitamin D-binding protein. The remaining nine peptides identified from protein spot 1 were also located in vitamin D-binding protein. The total sequence coverage was 32.70%.

**Table 3:** Results from mass spectrometry of six different spots in the AH of myopia patients.

Spot number	Protein description	Identified peptide	MH+	Δ Cn	Xcorr
1	Vitamin D-binding protein precursor	K.LPDATPTELAK.L	1156.31	0.38	3.67
		K.SLGECCDVEDSTTCFNAK.G	2094.24	0.36	2.72
		K.AKLPDATPTELAK.L	1355.56	0.47	2.83
		K.EVVSLTEACCAEGADPDCYDTR.T	2519.69	0.57	3.14
		K.GQELCADYSENTFTEYK.K	2056.15	0.38	4.6
		K.FPSGTFEQVSQLVK.E	1567.77	0.5	3.63
		K.SYLSMVGSCCTSASPTVCFLK.E	2356.75	0.39	3.17
		R.KFPSGTFEQVSQLVK.E	1695.94	0.36	4.56
		K.EYANQFMWEYSTNYGQAPLSLLV SYTK.S	3205.54	0.36	4.13
		K.VPTADLEDVLPLAEDITNILSK.C	2367.68	0.53	3.83
		R.KAADDTWEPFASGK.T	1523.63	0.55	4.11
		K.AADDTWEPFASGK.T	1395.46	0.46	3.65
		R.GSPAINVAVHVFR.K	1367.58	0.37	2.59
2	transthyretin	K.ALGISPFHEHAEVVFTANDSGPR.R	2452.67	0.42	5.41
		K.TSESGELHGLTTEEEFVEGIYK.V	2456.6	0.56	4.27
		R.RYTIAALLSPYSYSTTAVVTNPK.E	2517.86	0.62	4.68
		R.YTIAALLSPYSYSTTAVVTNPKE.	2490.79	0.49	4.9
3	albumin precursor	K.KVPQVSTPTLVEVSR.N	1640.91	0.53	5.23
		K.FQNALLVR.Y	961.14	0.18	2.82
		K.VPQVSTPTLVEVSR.N	1512.73	0.5	3.69
		K.ADDKETCFAEEGKK.L	1628.71	0.49	4.9
		K.LVAASQAALGL.-	1014.2	0.59	3.39
		K.CCAAADPHECYAK.V	1553.63	0.36	4.29
		R.RPCFSALEVDETYVPK.E	1912.12	0.32	3.87
		K.QNCELFEQLGEYK.F	1658.78	0.56	4.37
		K.EFNAETFTFHADICTLSEK.E	2261.42	0.62	5.65
		K.AVMDDFAAFVEK.C	1343.53	0.5	3.94
		K.AVM@DDFAAFVEK.C	1359.53	0.51	3.36
		K.ADDKETCFAEEGK.K	1500.54	0.39	3.22
		K.VFDEFKPLVEEPQNLIK.Q	2046.35	0.53	5.36
		K.DVFLGM@FLYEYAR.R	1640.88	0.53	4.04
		K.DVFLGMFLYEYAR.R	1624.88	0.46	4.25
		R.MPCAEDYLSVVLNQLCVLHEK.T	2519.88	0.49	6.18
		K.CCTESLVNR.R	1139.23	0.43	3.35
4	albumin precursor	K.LVNEVTEFAK.T	1150.31	0.54	3.63
		K.KVPQVSTPTLVEVSR.N	1640.91	0.52	3.65
		K.VPQVSTPTLVEVSR.N	1512.73	0.52	3.72
		K.QNCELFEQLGEYK.F	1658.78	0.6	4.29
		K.YICENQDSISSK.L	1444.52	0.53	3.59
		K.VFDEFKPLVEEPQNLIK.Q	2046.35	0.52	5.28
		K.YICENQDSISSK.L	1444.52	0.51	3.8
		K.SHCIAEVENDEMPADLPSLAADFV ESK.D	2976.21	0.46	5.73
		K.ALVLIAFAQYLQQCPFEDHVK.L	2491.86	0.59	5.46
5	albumin precursor	K.VFDEFKPLVEEPQNLIK.Q	2046.35	0.53	4.33
		K.KVPQVSTPTLVEVSR.N	1640.91	0.54	2.83
6	albumin precursor	K.FQNALLVR.Y	961.14	0.25	2.86
		K.KVPQVSTPTLVEVSR.N	1640.91	0.54	4.28
		K.VPQVSTPTLVEVSR.N	1512.73	0.46	3.14
		R.RPCFSALEVDETYVPK.E	1912.12	0.26	3.64
		K.AVM@DDFAAFVEK.C	1359.53	0.57	3.52
		K.QNCELFEQLGEYK.F	1658.78	0.61	4.33
		K.VFDEFKPLVEEPQNLIK.Q	2046.35	0.51	5.29

Results from mass spectrometry of six spots identified by two-dimensional gels to occur in the aqueous humor of patients with myopia (n=5) in an amount at least twice that observed in controls (n=5). The spots were derived from vitamin D-binding protein(No 1), transthyretin(No 2) and albumin(No 3-6).

Using the same methods, protein spot 2, with a significant increase in patients, was identified as transthyretin with sequence coverage of 65.31%. Significantly increased protein spots 3, 4, 5, and 6 were all identified as albumins with sequence coverages of 29.72%, 18.90%, 5.25%, and 13.30% respectively. When compared with the 2DE results, protein spots 3 and 4 had similar molecular weights but different pI values, suggesting that the difference between spots 3 and 4 may be caused by the posttranslational modification of albumin such as phospholation. Protein spots 5 and 6 had lower molecular weights (spot 5=40.0 Da and spot 6=33.5 Da) with a higher pI value than spot 3 and 4, indicating that protein spots 5 and 6 were the hydrolyzed peptides of albumin at different degrees. However, in the present study, the enzymes, which result in the posttranslation modifications and albumin degradation, remained undetected.

## Discussion

It has been demonstrated that various tissues of the eye are involved in the pathogenesis of high myopia, which is associated with active scleral elongation and tissue remodeling. The choroidal and scleral layers of the eye comprise the two principal targets of growth regulation. The choroid thickens in response to myopia defocus [17], the lymphatic vessels demarcate the scleral side of the choroids, and the major choroidal blood vessels are constricted [18]. The development of high myopia is associated with reduced scleral collagen accumulation, scleral thinning, and loss of scleral tissue as demonstrated in both human and animal models [19,20]. Numerous proteins are involved, including structural components (such as collagen and proteoglycans [21]), enzymes (such as the matrix metalloproteinases, MMPs), and tissue inhibitors of metalloproteinases (TIMPs) [22]. Evidence for local growth control necessarily implicated the retina as the source of growth-modulating signals. Since retinoic acid could play a role in the visual control of postnatal eye growth in primates possibly by inducing changes in the scleral extracellular matrix associated with increasing eye size, the excessive enlargement of the eye seen in myopia is known to be regulated, in part, by retinal neurotransmitters [23]. The retinal pigment epithelial (RPE) may relay signals between the retina and adjacent choroidal and scleral layers. Three retinal neurotransmitters (dopamine, vasoactive intestinal peptide, and glucagons that have been linked to eye growth regulation) are likely to affect RPE ion and fluid transportation. The concentration and activity of growth factors acting on the choroids and sclera might also be modulated by fluid transportation across the RPE [24].

Most of the recent insights regarding high myopia have come from studies on animal models. In addition, these studies typically focus on the retina, choroid, and sclera. In this study, we directed our attention to proteins of the AH. AH can be collected directly from patients, and the AH proteins might manifest discrete changes in patients with high myopia. In addition, it has been demonstrated that the total transforming growth factor (TGF) concentration of the AH shows slight changes as associated with axial length [25]. However, few studies have been involved with an examination of changes in other AH proteins in myopia. Therefore, it was considered of importance to study the changes of AH proteins that are present in patients. Such information might offer new insights to elucidate the mechanism of high myopia and identify potential biomarkers of this condition.

In this study, we compared AH proteins between patients with high myopia and non-myopic cataract (as the control). The patterns of 2D electrophoresis gels in high myopia were different from controls, which indicated that the protein content in the AH changes with myopia development. However, the origin and function of AH proteins is still unknown. Results from previous studies have implicated that AH proteins could activate signaling cascades, which subsequently regulate cellular functions including mitosis, differentiation, motility, and apoptosis. Such proteins play a vital role in corneal wound healing, mediating the proliferation of epithelial and stromal tissue, and affect remodeling of the extracellular matrix (ECM). Moreover, previous studies have demonstrated that AH could enter the anterior choroidal fluid. When taken together, the findings from these reports suggest that alterations in proteins could contribute to the pathologic changes and complications of myopia by at least two ways: by directly affecting the ocular anterior segment or through effects upon choroidal tissues. Further investigations are necessary to understand the exact relationship between AH changes and high myopia.

Vitamin D-binding protein (DBP) is a multi-functional plasma protein with many important functions. These include transportation of vitamin D metabolites, control of bone development, binding of fatty acids, sequestration of actin, and a range of less well defined roles involved with modulating immune and inflammatory responses [26]. However, the potential role of DBP functions in ocular tissues remains unclear. To our knowledge, the present findings are the first report indicating that DBP is related to high myopia. Previous studies have demonstrated that Apolipoprotein A1 (ApoA1) and DBP are upregulated in certain diseases, including procedures involved with transplantation of bone marrow cells [27] and in hypercholesterolemia [28]. Moreover, it has been suggested that DBP might be involved in the pathophysiology of the ApoA1 pathway in some diseases. Such changes in ApoA1 and DBP can have significant implications related to myopia. In a recent study, Bertrand et al. [29] reported that ApoA1 was one of the “STOP” signals for myopia. Hirai et al. [30] reported that genetic variations of DBP were associated with insulin resistance. Since elevated DBP would increase insulin tolerance and decrease insulin-like growth factor-1 (IGF-1) production [31] and it was demonstrated that IGF-1 could increase myopia [32], a clear link between DBP and myopia can be appreciated. Additionally, TGF-β is a transcriptional regulator of 1,25D(3)-membrane-associated, rapid-response steroid-binding (MARRS) expression via a functional Smad 3 element, and there exists cross-talk with the non-classical 1,25(OH)(2)D(3)-stimulated pathway [32]. As TGF-β isoforms are involved in the control of scleral remodeling during myopia development, the early alterations in TGF-β expression levels may reflect a role for these cytokines in mediating the retinoscleral signal that controls myopic eye growth [33]. In our study, all cases were adults where axial elongation should be stable. Therefore, we propose that DBP might be involved in the STOP pathway of ApoA1 to balance the possible “GROW” signals.

We also observed a significant increase of transthyretin (TTR) in our patients. Since it has been reported that TTR could be synthesized by ciliary pigment epithelium [34], this suggests that the TTR upregulation in myopic AH might be due to the alteration of ocular growth. Interestingly, TTR exists in relatively high concentrations in body fluids like serum and cerebrospinal fluid. Moreover, TTR has been associated with diseases such as Alzheimer disease [35] and type 2 diabetes [36]. Our current results suggest that TTR may also be associated with myopia.

In our experiments, additional spots were identified, which might be derived from albumin, according to the characterization and previous reports [15]. However, the albumin fragments obtained were different in pI and/or molecular weights. While we do not have a complete explanation for this finding, we speculate that it could be due to the degradation of pathways of albumin in AH from these patients.

In conclusion, the results of the present study revealed that the proteomic composition of AH was significantly different between the high myopia and control conditions. The proteins identified could function as STOP signal candidates and serve as potential biomarkers for myopia development.
